# Apoptosis signal-regulating kinase 1 exhibits oncogenic activity in pancreatic cancer

**DOI:** 10.18632/oncotarget.12090

**Published:** 2016-09-17

**Authors:** Youguang Luo, Siqi Gao, Ziwei Hao, Yang Yang, Songbo Xie, Dengwen Li, Min Liu, Jun Zhou

**Affiliations:** ^1^ Institute of Biomedical Sciences, College of Life Sciences, Key Laboratory of Animal Resistance Biology of Shandong Province, Key Laboratory of Molecular and Nano Probes of the Ministry of Education, Shandong Normal University, Jinan 250014, China; ^2^ State Key Laboratory of Medicinal Chemical Biology, College of Life Sciences, Nankai University, Tianjin 300071, China

**Keywords:** pancreatic cancer, ASK1, kinase activity, cell proliferation, cell migration

## Abstract

Pancreatic cancer has an extremely grim prognosis, with an overall 5-year survival rate less than 5%, as a result of its rapid metastasis and late diagnosis. To combat this disease, it is crucial to better understand the molecular mechanisms that contribute to its pathogenesis. Herein, we report that apoptosis signal-regulating kinase 1 (ASK1) is overexpressed in pancreatic cancer tissues and that its expression correlates with the histological grade of pancreatic cancer. The expression of ASK1 is also elevated in pancreatic cancer cell lines at both protein and mRNA levels. In addition, ASK1 promotes the proliferation and stimulates the tumorigenic capacity of pancreatic cancer cells. These functions of ASK1 are abrogated by pharmacological inhibition of its kinase activity or by introduction of a kinase-dead mutation, suggesting that the kinase activity of ASK1 is required for its role in pancreatic cancer. However, the alteration of ASK1 expression or activity does not significantly affect the migration or invasion of pancreatic cancer cells. Collectively, these findings reveal a critical role for ASK1 in the development of pancreatic cancer and have important implications for the diagnosis and treatment of this malignancy.

## INTRODUCTION

Pancreatic cancer is a highly malignant disease that is a leading cause of cancer-associated deaths. Less than 5% of patients survive five years after diagnosis. Due to the late diagnosis and frequent metastasis, approximately 90% of pancreatic cancer patients cannot undergo curative surgical intervention [1, 2]. Several tumor suppressor genes and oncogenes have been shown to be mutated in pancreatic cancer; for example, KRAS2, TP53, SMAD4, and other tumor-associated genes have been demonstrated to contribute to the pathogenesis of pancreatic cancer by promoting the expression of downstream genes and disrupting the cell cycle [3]. Despite this knowledge, a complete understanding of the underlying mechanisms that drive pancreatic cancer remains elusive. Furthermore, we currently lack biomarkers to diagnose this disease at an early stage and, thereby, improve the survival rate.

Apoptosis signal-regulating kinase 1 (ASK1), also known as mitogen-activated protein kinase kinase kinase 5, is a serine/threonine kinase that activates the c-Jun N-terminal kinase (JNK)- and p38-associated signaling pathways [4, 5]. ASK1 is primarily activated by cytotoxic stresses, such as lipopolysaccharide, reactive oxygen species, and endoplasmic reticulum stress [6–10]. ASK1 has been shown to be involved in many physiological processes, including apoptosis and cell proliferation, as well as in pathological processes, such as cardiovascular diseases and neurodegenerative diseases [11–14]. Recently, ASK1 has been linked to the development of several cancers. In gastric cancer, ASK1 and cyclin D1 were shown to form a positive feedback loop that promotes tumorigenesis [15]. In skin cancer, ASK1 promotes the proliferation of cancer cells by stimulating cytokine secretion [16]. In liver cancer, ASK1 acts as a tumor suppressor by promoting apoptosis and enhancing the expression of p21 [17]. Thus, ASK1 appears to play varied tissue-dependent roles in cancer development.

The role of ASK1 in pancreatic cancer is currently unknown. Although it is widely expressed, ASK1 is particularly abundant in the pancreas [5], where it has been shown to be involved in the development of diabetes [18]. Thus, the goal of this study was to define the potential role of ASK1 in the pathogenesis of pancreatic cancer.

## RESULTS

### ASK1 is overexpressed in pancreatic cancer

**Figure 1:**
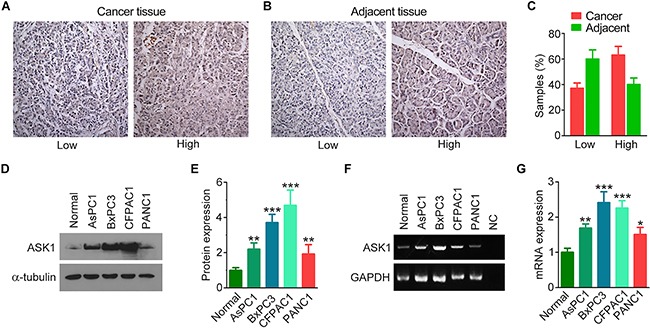
ASK1 is overexpressed in pancreatic cancer **A, B.** Representative images showing immunohistochemical staining of ASK1 expression in pancreatic cancer tissues (A) and adjacent tissues (B). **C.** Quantification of ASK1 expression in pancreatic cancer tissues (n = 19) and adjacent tissues (n = 10). **D, E.** Western blot analysis (D) and quantification (E) of ASK1 expression in normal pancreatic epithelial cells and pancreatic cancer cell lines. Experiments were repeated 3 times. **F, G.** RT-PCR analysis (F) and quantification (G) of ASK1 mRNA expression in normal pancreatic epithelial cells and pancreatic cancer cell lines. NC, negative control (i.e., without RNA). Experiments were repeated 3 times. Error bars represent mean ± SEM. *p < 0.05; **p < 0.01; ***p < 0.001.

To investigate whether ASK1 plays a role in pancreatic cancer tumorigenesis, we compared the expression of ASK1 in pancreatic cancer tissues and adjacent tissues by immunohistochemistry (Figure 1A, 1B). All of the samples analyzed were positive for ASK1 staining. Intriguingly, more than 60% of cancer tissues showed high expression of ASK1, while only 40% of adjacent tissues showed high expression (Figure 1C). This differential staining pattern suggests that ASK1 is overexpressed in pancreatic cancer. To further explore this hypothesis, we compared ASK1 expression in normal pancreatic epithelial cells and four pancreatic cancer cell lines, including AsPC1, BxPC3, CFPAC1, and PANC1. Western blotting revealed that ASK1 was expressed at higher levels in all of these pancreatic cancer cell lines compared to normal cells (Figure 1D, 1E). Analysis of ASK1 mRNA expression revealed similar results (Figure 1F, 1G). Together, these data indicate that ASK1 is overexpressed in pancreatic cancer and may play a role in the development of this disease.

### ASK1 expression correlates with clinicopathological variables

**Figure 2:**
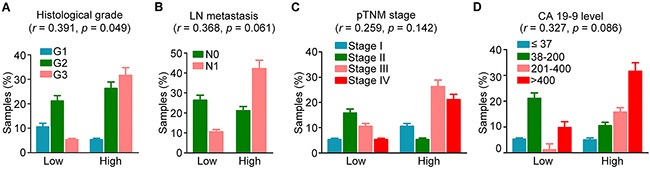
Correlation of ASK1 expression with clinicopathological variables of pancreatic cancer ASK1 expression was examined by immunohistochemical staining as shown in Figure 1. The correlation between ASK1 expression and histological grade **A.** lymph node (LN) metastasis **B.** pathological tumor node metastasis (pTNM) stage **C.** and carbohydrate antigen 19-9 (CA19-9) level **D.** was analyzed by the Spearman's rank correlation test (n = 19). The correlation coefficient (*r*) and *p* values were then calculated. Error bars represent mean ± SEM.

To gain further insight into the role of ASK1 in pancreatic cancer pathogenesis, we analyzed the potential association between ASK1 expression and clinicopathological parameters related to disease progression. Although no significant correlation was identified between ASK1 expression and lymph node (LN) metastasis, pathological tumor node metastasis (pTNM) stage, or the level of carbohydrate antigen 19-9 (CA19-9), the standard serum marker for pancreatic cancer, ASK1 expression significantly correlated with the histological grade of pancreatic cancer (Figure 2A-2D). These results provide further evidence supporting a role for ASK1 in the pathogenesis of pancreatic cancer.

### ASK1 depletion impairs the proliferation of pancreatic cancer cells

**Figure 3:**
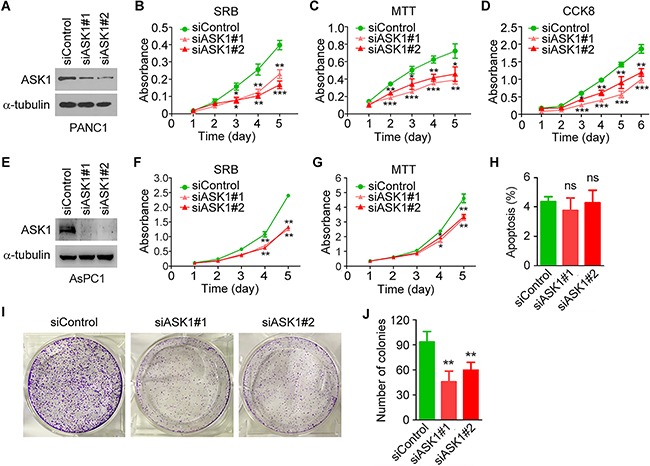
ASK1 knockdown impairs pancreatic cancer cell proliferation **A.** Western blot analysis of ASK1 and α-tubulin expression in PANC1 cells transfected with control or ASK1-targeted siRNAs. **B**-**D.** PANC1 cells were transfected with control or ASK1-targeted siRNAs, and cell proliferation was examined by SRB (B), MTT (C), and CCK8 (D) assays. **E.** Western blot analysis of ASK1 and α-tubulin expression in AsPC1 cells transfected with control or ASK1-targeted siRNAs. **F**, **G.** AsPC1 cells were transfected with control or ASK1-targeted siRNAs, and cell proliferation was examined by SRB (F) and MTT (G) assays. **H.** PANC1 cells were transfected with control or ASK1-targeted siRNAs, and the percentage of apoptotic cells was examined by staining with annexin V-FITC and propidium iodide followed by flow cytometry. **I**, **J.** Representative images from colony formation assays (I) and quantification of colonies (J) derived from PANC1 cells transfected with the indicated siRNAs. Error bars represent mean ± SEM. *p < 0.05, **p < 0.01, ***p < 0.001; ns, not significant.

To gain mechanistic insight into the role of ASK1 in pancreatic cancer, we used ASK1-targeted siRNAs to knock down the expression of ASK1 in PANC1 cells (Figure 3A). We then analyzed the effect of ASK1 siRNAs on cell proliferation using sulforhodamine B (SRB) staining assay. As shown in Figure 3B, PANC1 cells treated with ASK1-targeted siRNAs proliferated more slowly than control cells. Similar results were obtained using 3-(4,5-dimethylthiazol-2-yl)-2,5-diphenyltetrazolium bromide (MTT) and cell counting kit-8 (CCK8) assays (Figure 3C, 3D). By SRB and MTT assays, we also found that siRNA-mediated knockdown of ASK1 expression significantly inhibited the proliferation of AsPC1 pancreatic cancer cells (Figure 3E-3G). By contrast, ASK1 siRNAs did not significantly affect the proliferation of normal pancreatic epithelial cells (Supplementary Figure S1). These results suggest that ASK1 is important for pancreatic cancer cell proliferation and that this effect is specific to malignant cells.

We then investigated whether ASK1 siRNAs affect apoptosis of pancreatic cancer cells. By staining cells with annexin V-FITC and propidium iodide followed by flow cytometry, we found that siRNA-mediated knockdown of ASK1 expression did not significantly affect apoptosis of PANC1 cells (Figure 3H and Supplementary Figure S2).

We next analyzed the role of ASK1 in tumorigenesis using colony formation assays. We found that siRNA-mediated ASK1 knockdown dramatically decreased the number of colonies formed from PANC1 cells (Figure 3I, 3J). These results suggest that ASK1 regulates the proliferation and tumorigenic capability of pancreatic cancer cells.

### The kinase activity of ASK1 is required for its regulation of pancreatic cancer cell proliferation

**Figure 4:**
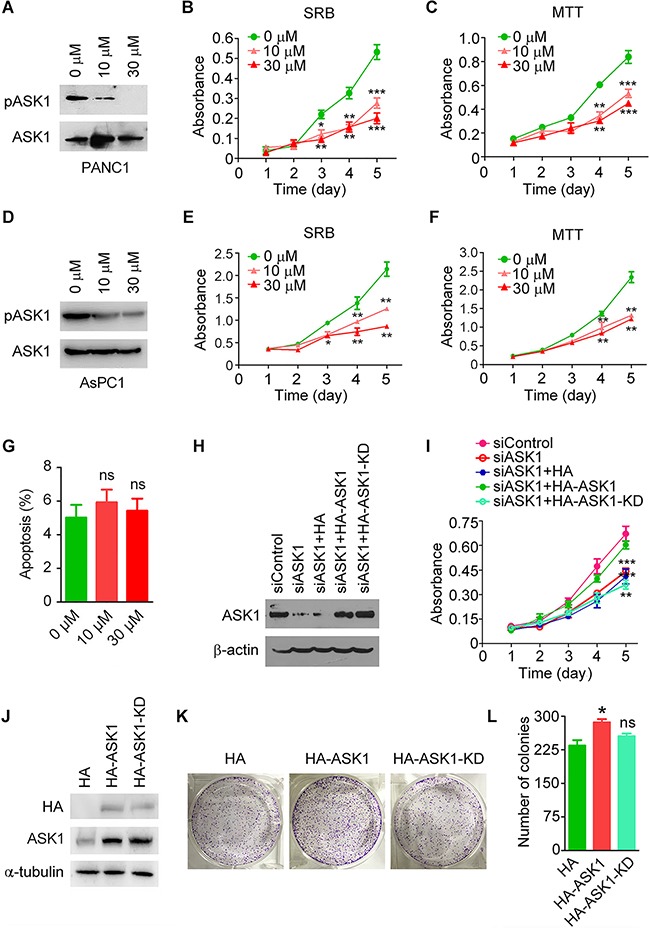
The kinase activity is required for ASK1 to regulate pancreatic cancer cell proliferation **A.** Western blot analysis of ASK1 phosphorylated at threonine 845 (pASK1) and total ASK1 in PANC1 cells treated with the indicated doses of the ASK1-specific inhibitor NQDI-1. **B, C.** PANC1 cells were treated with NQDI-1, and cell proliferation was analyzed by SRB (B) and MTT (C) assays. **D.** Western blot analysis of pASK1 and total ASK1 in AsPC1 cells treated with the indicated doses of NQDI-1. **E, F.** AsPC1 cells were treated with NQDI-1, and cell proliferation was analyzed by SRB (E) and MTT (F) assays. **G.** PANC1 cells were treated with the indicated doses of NQDI-1, and the percentage of apoptotic cells was examined by staining with annexin V-FITC and propidium iodide followed by flow cytometry. **H, I.** Western blot analysis of ASK1 and β-actin expression (H) and examination of cell proliferation by SRB assay (I) in PANC1 cells transfected with the indicated siRNAs and plasmids. KD, kinase dead. **J, K.** PANC1 cells overexpressing HA, HA-ASK1, or HA-ASK1-KD were subjected to Western blotting (J) and colony formation assays (K). **L.** Quantification of colonies derived from PANC1 cells transfected with the indicated plasmids. Error bars represent mean ± SEM. *p < 0.05, **p < 0.01, ***p < 0.001; ns, not significant.

To investigate whether the kinase activity of ASK1 is required for its regulation of PANC1 cell proliferation, we treated PANC1 cells with NQDI-1, a potent ASK1 inhibitor [19]. In agreement with the finding that ASK1 undergoes autophosphorylation at threonine 845 [20], inhibition of ASK1 activity by NQDI-1 suppressed the level of ASK1 phosphorylation in a dose-dependent manner (Figure 4A). By SRB and MTT assays, we found that NQDI-1 treatment significantly inhibited PANC1 cell proliferation in a dose-dependent manner (Figure 4B, 4C). By SRB and MTT assays, we also found that NQDI-1 treatment remarkably suppressed the proliferation of AsPC1 cells (Figure 4D-4F), but not normal pancreatic epithelial cells (Supplementary Figure S1). By flow cytometry, we further found that inhibition of ASK1 activity by NQDI-1 did not significantly affect apoptosis of PANC1 cells (Figure 4G and Supplementary Figure S2).

To confirm our findings about the effect of ASK1 on pancreatic cancer cell proliferation, we performed rescue experiments. We found that overexpression of HA-ASK1, but not HA or HA-ASK1-KD (a kinase dead mutant of ASK1, i.e., K709R), rescued the decreased cell proliferation of PANC1 cells caused by ASK1 depletion (Figure 4H, 4I). In the colony formation assay, we also found that overexpression of HA-ASK1, but not HA or HA-ASK1-KD, increased colony number (Figure 4J-4L). Together, these results indicate that the kinase activity of ASK1 is required for its role in the modulation of cell proliferation.

### ASK1 depletion or overexpression does not affect pancreatic cancer cell migration or invasion

**Figure 5:**
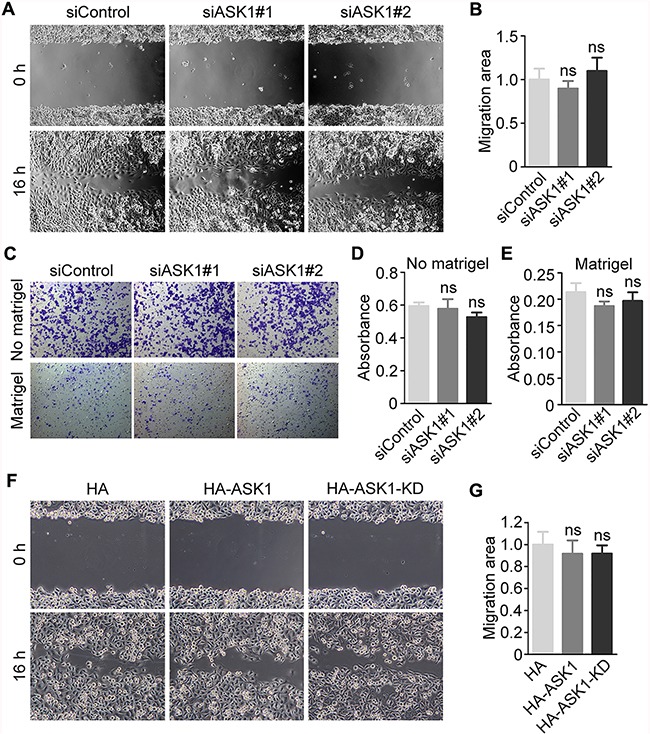
Effect of ASK1 on the motility of pancreatic cancer cells **A.** PANC1 cells were transfected with the indicated siRNAs, and cell migration was examined by wound healing assays. **B.** Quantification of (A). The relative migration area at 16 hours after scratch creation was measured. **C.** Transwell assays were performed using PANC1 cells transfected with control or ASK1 siRNAs in the absence or presence of matrigel on the upper surface of the transwell filters. **D**, **E.** Migratory (D) and invasive (E) abilities of pancreatic cancer cells were quantified based on experiments shown in the upper and lower panels, respectively in (C). **F, G.** PANC1 cells were transfected with plasmids expressing HA, HA-ASK1, or HA-ASK1-KD, and cell migration was analyzed using wound healing assays. Error bars represent mean ± SEM. ns, not significant.

To characterize the potential role of ASK1 in pancreatic cancer progression, we analyzed the effect of ASK1 depletion on the migration of PANC1 cells. Analysis of the extent of wound healing in a scratch assay revealed no significant differences between PANC1 cells treated with control and ASK1-specific siRNAs (Figure 5A, 5B). Similar results were obtained using transwell migration assays in the absence or presence of matrigel (Figure 5C-5E). Consistent with these observations, overexpression of HA-ASK1 or HA-ASK1-KD had no obvious effect on PANC1 cell migration in the scratch assay (Figure 5F, 5G). These results suggest that the role of ASK1 in pancreatic cancer is not likely associated with changes in the migration and invasion of cancer cells.

### ASK1 knockdown impairs pancreatic tumor growth in mice

**Figure 6:**
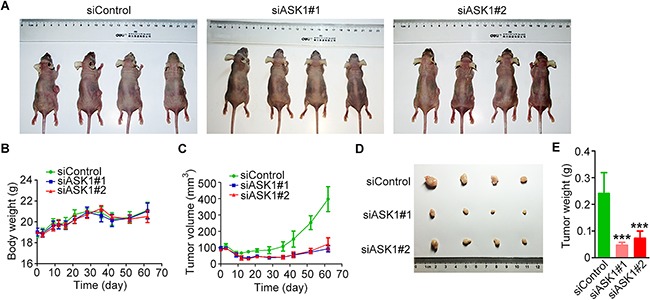
ASK1 promotes pancreatic tumorigenesis in mice **A.** Representative images of nude mice bearing tumors derived from PANC1 cells pretreated with the indicated siRNAs. **B**, **C.** Body weight (B) and tumor volume (C) were measured over a two-month period. **D**, **E.** After mice were sacrificed, tumors were dissected and photographed (D), and tumor mass was measured (E). Error bars represent mean ± SEM. ***p < 0.001.

To explore the role of ASK1 in pancreatic cancer cell proliferation in vivo, we analyzed the growth of tumors derived from PANC1 cells treated with control or ASK1-targeted siRNAs (Figure 6A). Analysis of the body weight of mice revealed no significant differences between mice bearing control or ASK1-depleted tumors over the course of the experiment (Figure 6B). Tumors derived from PANC1 cells treated with ASK1-targeted siRNAs grew more slowly than those from cells treated with the control siRNA (Figure 6C). Analysis of tumor mass revealed that tumors in the control group had an average mass of 0.23 g, while those from the ASK1 knockdown groups weighed less than 0.1 g on average (Figure 6D, 6E). These data confirm that ASK1 plays an important role in pancreatic tumorigenesis in vivo.

## DISCUSSION

Pancreatic cancer has a high mortality rate due to its frequent late diagnosis and lack of effective treatments. Currently, our knowledge of the mechanisms underlying this disease is very limited, and novel therapeutic targets and diagnostic markers are urgently needed. Herein, we report a novel role for ASK1 in pancreatic cancer development. ASK1 is overexpressed in pancreatic cancer cells, where it promotes cell proliferation. This role in tumorigenesis suggests that ASK1 acts as an oncogene in the development of pancreatic cancer. Moreover, the kinase activity of ASK1 is required for its role in pancreatic cancer cell proliferation, indicating that ASK1 directly or indirectly regulates the phosphorylation of downstream effectors that promote the proliferation of pancreatic cancer cells.

Interestingly, in gastric cancer ASK1 promotes the expression of cyclin D1 via phosphorylation of JNK, and cyclin D1 increases ASK1 expression by activating the Rb/E2F pathway [15]. Thus, ASK1 promotes gastric cancer development through an autoregulatory, positive feedback loop. We found that siRNA-mediated knockdown of ASK1 expression in PANC1 cells inhibited the expression of cyclin E, but not cyclin D (Supplementary Figure S3), suggesting that ASK1 might promote pancreatic cancer development by upregulating cyclin E. Additionally, inflammation might be involved in the tumor-promoting effects of ASK1 in pancreatic cancer. Recent studies show that ASK1 and its downstream substrates, such as p38, can promote tumorigenesis through the production of inflammatory cytokines [16, 21]. The detailed mechanisms that underlie the role of ASK1 in pancreatic cancer require additional investigation in future studies.

In the present study, our data show that ASK1 plays an important role in pancreatic cancer cell proliferation, but has no overt effect on cell migration. This observation is not surprising in light of the fact that ASK1 activity has not often been reported to be associated with cell migration or metastasis. In fact, the role of ASK1 in the regulation of cell motility is controversial. One study demonstrates that the migration of vascular smooth muscle cells from ASK1 knockout mice is significantly attenuated compared to those from wild-type mice [22]. However, another study shows that ASK1 depletion stimulates breast cancer cell migration [23]. Therefore, ASK1-mediated regulation of cell motility might be cell type-specific and depend on the cell context.

Different roles have also been reported for ASK1 in cancer development. In gastric cancer, ASK1 promotes tumor development, while in liver cancer and colon cancer, ASK1 acts as a tumor suppressor [15–17, 24]. With respect to pancreatic cancer, a recent study reported that ASK1 is involved in the anti-cancer activity of capsaicin through the promotion of apoptosis [25], while another study found that ASK1 had no overt effect on the proliferation of BxPC3 or AsPC1 pancreatic cancer cells [15]. Our results show that ASK1 promotes the proliferation of PANC1 and AsPC1 pancreatic cancer cells in vitro and stimulates tumor growth in mice. To better understand these seemingly conflicting results, more information is needed about the detailed mechanisms associated with the role of ASK1 in cancer development.

As a result of structural studies, potent inhibitors of ASK1 have been developed and characterized. The therapeutic efficacy of ASK1 inhibitors has been explored in the context of many diseases, including gastric cancer, neurodegenerative disorders, and ischemia reperfusion injury [26–28]. In this study, we found that treatment with an ASK1 inhibitor dramatically suppressed the proliferation of PANC1 pancreatic cancer cells, suggesting that ASK1 inhibitors may have value for the treatment of pancreatic cancer. Taken together, our results suggest that ASK1 has potential as a therapeutic target for pancreatic cancer and could also serve as a biomarker for diagnosis of the disease.

## MATERIALS AND METHODS

### Ethics statement

Investigation has been conducted in accordance with the ethical standards according to the Declaration of Helsinki and the national and international guidelines, and has been approved by the authors' institutional review board.

### Antibodies, chemicals, siRNAs and plasmids

Antibodies against β-actin and α-tubulin (Sigma-Aldrich), ASK1 phosphorylated at threonine 845 (pASK1), ASK1, cyclin D, and cyclin E (Abcam), and horseradish peroxidase-conjugated secondary antibodies (Amersham Biosciences) were obtained from the indicated sources. SRB, MTT, and NQDI-1 were purchased from Sigma-Aldrich. Control siRNA (5′-CGUACGCGGAAUACUUCGA-3′) and ASK1 siRNAs (#1: 5′-GCACUCCU-UCAUCGAGCU-3′; #2: 5′-GGUAUACAUGAGUGGAAUU-3′) were synthesized by Ribo Bio. The mammalian expression plasmid for HA-ASK1 was constructed by cloning ASK1 cDNA into the pCMV-HA vector. The HA-ASK1-KD mutant was generated by site-directed mutagenesis.

### Cell culture and transfection

Cells were cultured in the DMEM medium supplemented with 10% fetal bovine serum at 37°C in a humidified atmosphere with 5% CO_2_. Plasmids and siRNAs were transfected into cells with TurboFect (Thermo Fisher Scientific) and Lipofectamine 2000 (Invitrogen), respectively.

### Western blotting

Protein samples were separated by SDS-PAGE and transferred onto polyvinylidenedifluoride membranes (Millipore). Then the membranes were blocked in 5% fat-free milk, and incubated sequentially with primary antibodies and horseradish peroxidase-conjugated secondary antibodies as described [29, 30]. Target proteins were visualized with enhanced chemiluminescence detection reagent according to the manufacturer's instructions (Pierce Biotechnology).

### Immunohistochemistry

Human pancreatic tissues, including 19 pancreatic cancer tissues and 10 adjacent tissues, were obtained from patients who underwent surgical resection at Shanxian Dongda Hospital. For the analysis of ASK1 expression in clinical samples, immunohistochemistry was performed as described previously [31, 32]. Briefly, paraffin-embedded tissue sections were cut, deparaffinized and rehydrated with xylene and graded alcohols. Antigen retrieval was performed in 5 mM citrate buffer. After inactivation of endogenous peroxidase with 3% H_2_O_2_, the sections were blocked with 2% bovine serum albumin and incubated with the primary antibody. Then the sections were incubated with biotinylated secondary antibody and streptavidin-biotin-peroxidase, and diaminobenzidine was used as a chromogen substrate. The sections were counterstained with hematoxylin. Protein expression was graded based on the intensity of staining and the percentage of stained cells as described [33, 34].

### Cell proliferation assays

Cells were seeded in 96-well plates with a density of 5,000 cells per well. For SRB staining, cells were fixed with 50% trichloroacetic acid, stained with 0.4% SRB and washed with 1% acetic acid. The protein-bound dye was extracted with 10 mM Tris-base, and the optical density at 490-nm wavelength was then detected as described [35]. The MTT and CCK8 assays were performed by using the Vybrant MTT cell proliferation assay kit (Thermo Fisher Scientific) and the cell counting kit-8 (Sigma-Aldrich), respectively, following the manufacturers' instructions.

### Colony formation assays

Cells were plated in 6-well plates with a density of 5,000 cells per well and grown for 2 weeks. Colonies were fixed with methanol and stained with 0.1% crystal violet. Photographs were then taken and the number of colonies was counted.

### Wound healing assays

Cells were grown in serum-free medium for 12 hours as confluent monolayers and mechanically scratched with a 20-μL pipette tip to create the wound. Cells were then washed with phosphate-buffered saline to remove the cell debris, and the complete culture medium containing 10% serum was added to allow for wound healing. Phase-contrast images of the wound were taken 16 hours later, and the migration areas of wound healing were measured and normalized by dividing the mean migration area of the control group as described previously [36, 37].

### Transwell assay

Cell migration and invasion in response to ASK1 depletion were carried out by the transwell assay as described previously [38, 39]. The upper surface of the transwell filter was coated with or without matrigel. Cells suspended in 200 μL of serum-free medium were added to the chamber, and the chamber was placed in a 24-well plate containing the complete medium. After 24 hours of incubation at 37°C, the filter was gently taken out and matrigel on the upper surface of the filter was removed by cotton swabs. Cells on the underside of the transwell filter were fixed with 4% paraformaldehyde for 30 minutes, stained with 0.1% crystal violet for 10 minutes, and photographed. The dye was then extracted with 10% acetic acid and quantified with a microplate reader at a wavelength of 562 nm.

### Examination of tumor growth in mice

Cells were injected subcutaneously into the right flanks of female athymic nude mice as described [40]. Tumor volume was measured with a vernier caliper and calculated with the following formula: V = π/6 X length X width^2^. The mice were then sacrificed and tumors were isolated from mice, photographed and weighed.

### Statistics

Analysis of statistical significance was performed by the Student's t-test for comparison between two groups and by the ANOVA test for multiple comparisons. Correlation coefficient was calculated by the Spearman's rank correlation test.

## SUPPLEMENTARY FIGURES


