# Supplementary material for: Apoptosis signal-regulating kinase 1 exhibits oncogenic activity in pancreatic cancer
Source: Oncotarget. 2016 Sep 17;7(46):75155–64. doi: 10.18632/oncotarget.12090 (PMC5342730; doi:10.18632/oncotarget.12090)
Supplement: Supplementary file 1 [file oncotarget-07-75155-s001.pdf]

Apoptosis signal-regulating kinase 1 exhibits oncogenic activity in pancreatic cancer

SUPPLEMENTARY FIGURES

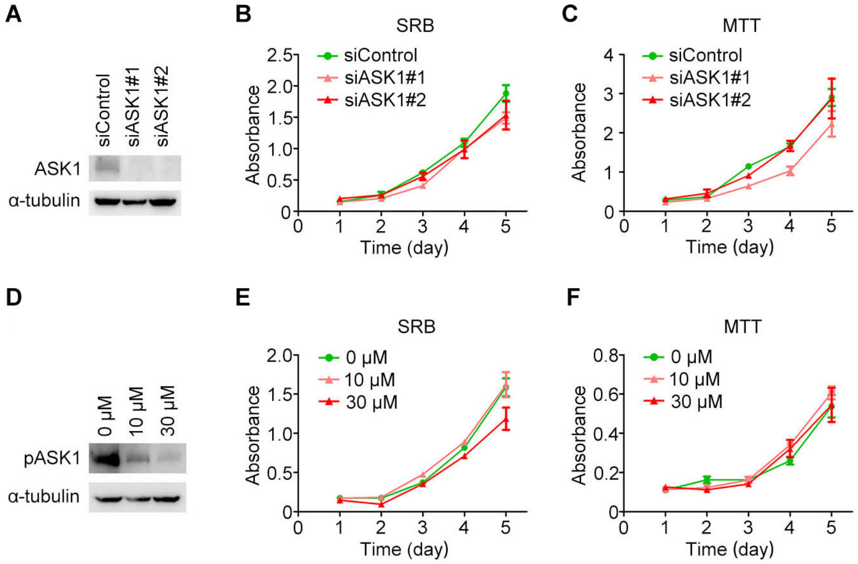

Supplementary Figure S1: Effects of ASK1 on the proliferation of normal pancreatic epithelial cells. A. Western blot analysis of ASK1 and α-tubulin expression in normal pancreatic epithelial cells transfected with control or ASK1-targeted siRNAs. **B, C.** Normal pancreatic epithelial cells were transfected with control or ASK1-targeted siRNAs, and cell proliferation was examined by SRB (B) and MTT (C) assays. **D.** Western blot analysis of ASK1 and α-tubulin expression in normal pancreatic epithelial cells treated with the indicated doses of NQDI-1. **E, F.** Normal pancreatic epithelial cells were treated with NQDI-1, and cell proliferation was examined by SRB (E) and MTT (F) assays.

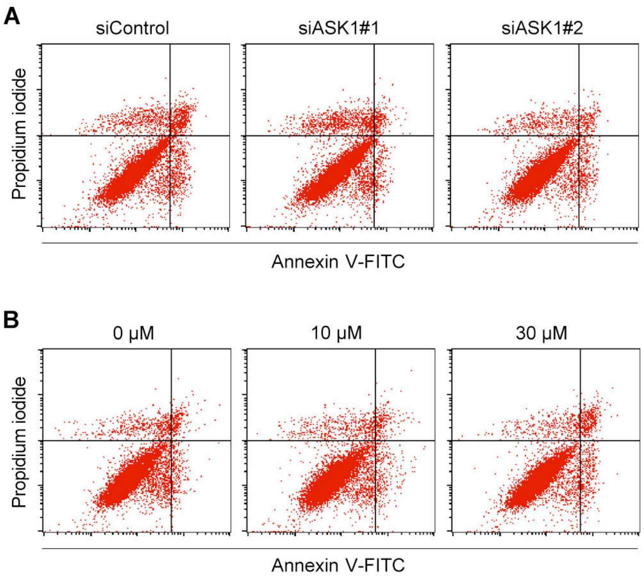

Supplementary Figure S2: Effects of ASK1 on apoptosis of pancreatic cancer cells. A. PANC1 cells were transfected with control or ASK1-targeted siRNAs, and the percentage of apoptotic cells was examined by staining with annexin V-FITC and propidium iodide followed by flow cytometry. **B.** PANC1 cells were treated with the indicated doses of NQDI-1, and the percentage of apoptotic cells was examined by staining with annexin V-FITC and propidium iodide followed by flow cytometry.

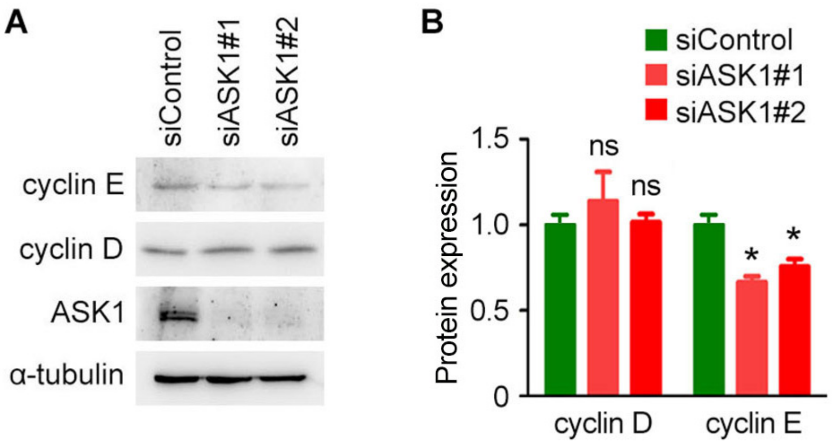

Supplementary Figure S3: Effects of ASK1 on the expression of cyclin D and cyclin E in pancreatic cancer cells. **A.** Western blot analysis of the indicated proteins in PANC1 cells transfected with control or ASK1-targeted siRNAs. **B.** Experiments were performed as in (A), and the expression of cyclin D and cyclin E was quantified. *$p < 0.05$; ns, not significant.
